# Association of Do-Not-Resuscitate Patient Case Mix With Publicly Reported Risk-Standardized Hospital Mortality and Readmission Rates

**DOI:** 10.1001/jamanetworkopen.2020.10383

**Published:** 2020-07-14

**Authors:** Benjamin D. Pollock, Jeph Herrin, Matthew R. Neville, Sean C. Dowdy, Pablo Moreno Franco, Nilay D. Shah, Henry H. Ting

**Affiliations:** 1 Department of Health Sciences Research, Mayo Clinic, Jacksonville, Florida; 2 Department of Quality, Experience, and Affordability, Mayo Clinic, Rochester, Minnesota; 3 Flying Buttress Associates, Charlottesville, Virginia; 4 Section of Cardiovascular Medicine, Yale University School of Medicine, New Haven, Connecticut; 5 Department of Obstetrics and Gynecology, Mayo Clinic, Rochester, Minnesota; 6 Department of Critical Care Medicine, Mayo Clinic, Jacksonville, Florida; 7 Department of Cardiovascular Diseases, Mayo Clinic, Rochester, Minnesota

## Abstract

**Importance:**

The Centers for Medicare and Medicaid Services’s (CMS’s) 30-day risk-standardized mortality rate (RSMR) and risk-standardized readmission rate (RSRR) models do not adjust for do-not-resuscitate (DNR) status of hospitalized patients and may bias Hospital Readmissions Reduction Program (HRRP) financial penalties and Overall Hospital Quality Star Ratings.

**Objective:**

To identify the association between hospital-level DNR prevalence and condition-specific 30-day RSMR and RSRR and the implications of this association for HRRP financial penalty.

**Design, Setting, and Participants:**

This cross-sectional study obtained patient-level data from the Medicare Limited Data Set Inpatient Standard Analytical File and hospital-level data from the CMS Hospital Compare website for all consecutive Medicare inpatient encounters from July 1, 2015, to June 30, 2018, in 4484 US hospitals. Hospitalized patients had a principal diagnosis of acute myocardial infarction (AMI), heart failure (HF), stroke, pneumonia, or chronic obstructive pulmonary disease (COPD). Incoming acute care transfers, discharges against medical advice, and patients coming from or discharged to hospice were among those excluded from the analysis.

**Exposures:**

Present-on-admission (POA) DNR status was defined as an *International Classification of Diseases, Ninth Revision* diagnosis code of V49.86 (before October 1, 2015) or as an *International Statistical Classification of Diseases and Related Health Problems, Tenth Revision* diagnosis code of Z66 (beginning October 1, 2015). Hospital-level prevalence of POA DNR status was calculated for each of the 5 conditions.

**Main Outcomes and Measures:**

Hospital-level 30-day RSMRs and RSRRs for 5 condition-specific cohorts (mortality cohorts: AMI, HF, stroke, pneumonia, and COPD; readmission cohorts: AMI, HF, pneumonia, and COPD) and HRRP financial penalty status (yes or no).

**Results:**

Included in the study were 4 884 237 inpatient encounters across condition-specific 30-day mortality cohorts (patient mean [SD] age, 78.8 [8.5] years; 2 608 182 women [53.4%]) and 4 450 378 inpatient encounters across condition-specific 30-day readmission cohorts (patient mean [SD] age, 78.6 [8.5] years; 2 349 799 women [52.8%]). Hospital-level median (interquartile range [IQR]) prevalence of POA DNR status in the mortality cohorts varied: 11% (7%-16%) for AMI, 13% (7%-23%) for HF, 14% (9%-22%) for stroke, 17% (9%-26%) for pneumonia, and 10% (5%-18%) for COPD. For the readmission cohorts, the hospital-level median (IQR) POA DNR prevalence was 9% (6%-15%) for AMI, 12% (6%-22%) for HF, 16% (8%-24%) for pneumonia, and 9% (4%-17%) for COPD. The 30-day RSMRs were significantly higher for hospitals in the highest quintiles vs the lowest quintiles of DNR prevalence (eg, AMI: 12.9 [95% CI, 12.8-13.1] vs 12.5 [95% CI, 12.4-12.7]; *P* < .001). The inverse was true among the readmission cohorts, with the highest quintiles of DNR prevalence exhibiting the lowest RSRRs (eg, AMI: 15.3 [95% CI, 15.1-15.5] vs 15.9 [95% CI, 15.7-16.0]; *P* < .001). A 1% absolute increase in risk-adjusted hospital-level DNR prevalence was associated with greater odds of avoiding HRRP financial penalty (odds ratio, 1.06; 95% CI, 1.04-1.08; *P* < .001).

**Conclusions and Relevance:**

This cross-sectional study found that the lack of adjustment in CMS 30-day RSMR and RSRR models for POA DNR status of hospitalized patients may be associated with biased readmission penalization and hospital-level performance.

## Key Points

**Question:**

Are risk-standardized mortality and readmission rates used in financial penalties and quality star ratings associated with the case mix of patients with do-not-resuscitate status in US hospitals?

**Findings:**

In this cross-sectional study of mortality cohorts comprising 4 884 237 inpatient encounters and readmission cohorts including 4 450 378 inpatient encounters, hospitals with a greater relative volume of patients with present-on-admission do-not-resuscitate status had statistically significantly higher 30-day risk-standardized mortality rates and statistically significantly greater odds of avoiding a Hospital Readmission Reduction Program financial penalty.

**Meaning:**

Results of this study suggest that risk-standardized hospital outcomes should control for do-not-resuscitate status to avoid potential biases in pay-for-performance programs and hospital quality rankings.

## Teaser

This cross-sectional study examines the association between rates of mortality and readmission used by Medicare to determine financial penalties and to rank hospital quality with the case mix of patients with do-not-resuscitate status at US hospitals.

## Introduction

The Centers for Medicare and Medicaid Services (CMS) has invested more than $130 million to develop, refine, and disseminate risk-standardized quality outcome models.^1^ These models are considered the criterion standard for risk standardization and have been adopted by professional societies in the US.^2,3^ Currently, the condition-specific 30-day risk-standardized mortality rate (RSMR) model is used under value-based purchasing to calculate financial reimbursement for 3 conditions: acute myocardial infarction (AMI), heart failure (HF), and pneumonia.^4^ Similarly, the condition-specific 30-day risk-standardized readmission rate (RSRR) model is used to establish Hospital Readmissions Reduction Program (HRRP) penalties,^5^ which are levied annually against most US hospitals, for 4 conditions: AMI, HF, chronic obstructive pulmonary disease (COPD), and pneumonia.^6^ In addition to the financial implications, 30-day RSMR and RSRR together account for 44% weight in the CMS Overall Hospital Quality Star Ratings. The 30-day RSMRs and RSRRs are adjusted for approximately 20 to 40 condition categories, which include common comorbidities such as cancer, diabetes, renal failure, and psychiatric disorders. However, a patient’s do-not-resuscitate (DNR) status is not included in risk adjustment despite the greater severity of illness and mortality risk among patients with DNR status present on admission (POA).^7,8,9,10^

Evidence is limited on the association between the prevalence of POA DNR status and the risk-standardized outcome rates at hospitals. Although hospital-level prevalence of POA DNR status varies, the DNR status of patients is documented reliably enough for inclusion in risk-standardization models and analyses, suggesting that adjustment for patient-level DNR status improves the predictive accuracy of 30-day RSMRs for AMI, HF, and pneumonia.^11,12,13^ To our knowledge, however, the association between hospital-level DNR prevalence and 30-day RSMRs and RSRRs has not been examined using national data from all Medicare-eligible US hospitals.

In this cross-sectional study, we assessed the variation in hospital-level POA DNR status across the condition-specific cohorts used in value-based purchasing reimbursement, HRRP penalty, and the CMS Overall Hospital Quality Star Ratings. Our aim was to identify the association between the prevalence of POA DNR status and condition-specific 30-day RSMRs and RSRRs. We also evaluated the implication of POA DNR status prevalence for the HRRP financial penalties.

## Methods 

This cross-sectional study was deemed exempt by the Mayo Clinic Institutional Review Board because the data used have been previously collected and deidentified. Informed consent was not required for this secondary research as contact with participants was not possible. We followed the Strengthening the Reporting of Observational Studies in Epidemiology (STROBE) reporting guideline.

### Data Source and Study Population

We obtained data from the 2015 to 2018 CMS Limited Data Set Inpatient Standard Analytical File, which includes 100% of Medicare fee-for-service inpatient encounters across 4484 US hospitals. The condition-specific cohorts for mortality were AMI, HF, stroke, pneumonia, and COPD. To form these condition-specific 30-day mortality cohorts, we applied *International Classification of Diseases, Ninth Revision* (*ICD-9*) codes (from July 1, 2015, to September 30, 2015) and *International Statistical Classification of Diseases and Related Health Problems, Tenth Revision* (*ICD-10*) codes (from October 1, 2015, to October 31, 2018) to the index admissions, adhering to the inclusion criteria specified in the CMS inpatient quality-reporting, condition-specific mortality documentation method,^14^ and we excluded incoming acute care transfers and patients who were discharged against medical advice. In the AMI, pneumonia, and HF mortality cohorts, we also excluded patients who were discharged alive within 1 day of admission who were not transferred to an acute care facility. We did not exclude patients who arrived from hospice or who were discharged to hospice.

The condition-specific cohorts for readmission were AMI, HF, pneumonia, and COPD. To form these condition-specific 30-day readmission cohorts, we followed the CMS inpatient quality-reporting 30-day readmission measures method^15^ and excluded all patients who died before discharge, were discharged against medical advice, or were discharged or transferred to an acute care facility. We did not exclude patients who arrived from hospice or were discharged to hospice. An important distinction is that CMS attributes 30-day readmissions to the discharging hospital, whereas 30-day mortalities are attributed to the admitting hospital. Thus, the transfer exclusion criteria for both RSMRs and RSRRs matched the exclusion criteria of the CMS method.

The 30-day mortality study population consisted of all consecutive Medicare inpatient encounters from July 1, 2015, through June 30, 2018, with a principal diagnosis of AMI, HF, stroke, pneumonia, or COPD. The 30-day readmission study population included all consecutive Medicare inpatient encounters from July 1, 2015, through June 30, 2018, with a principal diagnosis of AMI, HF, pneumonia, or COPD. This period matched the data period used for the 30-day RSMRs and RSRRs publicly reported on the CMS Hospital Compare website as of August 2019.

To ascertain the HRRP penalty status (yes or no) during fiscal years 2016 to 2018, we downloaded the Inpatient Pay-for-Performance Final Impact Summary File from the Advisory Board,^16^ which provided annual HRRP penalty status by hospital. Any hospital that was not penalized at any time during the 3 fiscal years (2016, 2017, or 2018) was considered to have the binary outcome of avoided HRRP penalization.

### Exposure and Outcomes

We defined DNR status as an *ICD-9* diagnosis code of V49.86 (before October 1, 2015) or an *ICD-10* diagnosis code of Z66 (beginning October 1, 2015) if the DNR order was POA per the corresponding POA indicator on the inpatient claim. Patients without a POA DNR status but who acquired DNR status after admission were included in the analysis. The outcomes were the hospital-level condition-specific 30-day RSMRs for AMI, HF, COPD, pneumonia, and stroke and the 30-day RSRRs for AMI, HF, COPD, and pneumonia as reported on the Hospital Compare website in August 2019. Briefly, the CMS calculates hospital-specific RSMRs and RSRRs by taking each hospital’s predicted vs expected (P/E) 30-day mortality or readmission rates from hierarchical mixed-effects risk-standardization models and multiplying these P/E ratios by the national mean 30-day mortality or readmission rate. We matched at the hospital level the values on the Hospital Compare website to the Inpatient Standard Analytical File data using the 6-digit CMS provider numbers. Hospitals without reported RSMR or RSRR data were excluded.

### Risk Adjustment

Using the Healthcare Cost and Utilization Project Elixhauser Comorbidity Software,^17^ version 3.7 and beta version, we assigned 29 binary comorbidity categories, known as Elixhauser comorbidities,^18^ to all patients on the basis of corresponding *ICD-9* or *ICD-10* diagnosis codes and Medicare severity diagnosis related group. We then used the Healthcare Cost and Utilization Project Comorbidity Index program to calculate the Elixhauser comorbidity index score from the weighted sum of these 29 comorbidity categories.^19,20^ The Elixhauser comorbidity index score is an integer ranging from –32 (lowest) to 99 (highest) comorbidity burden.

### Association Between DNR Status and Comorbidity Burden

We hypothesized that DNR status would be associated with a patient’s comorbidity burden. However, we also wanted to assess the shape of this association to ensure that an unexpected decline in DNR status did not occur at the sickest or healthiest patient extremes, which might suggest systematic bias in documentation. To test this hypothesis, we estimated a logistic regression model by pooling the patient-level data from the 5 condition-specific 30-day mortality cohorts, with DNR status as the dependent variable regressed on Elixhauser comorbidity index and age (both modeled using 5-knot restricted cubic spline functions)^21^ and with a condition indicator as a third independent variable and an interaction term between the condition and the Elixhauser comorbidity index score. We visually assessed the association between estimated probability of DNR prevalence (y-axis) and comorbidity burden (x-axis) (eFigure 1 in the Supplement).

### Variation in Hospital-Level DNR Prevalence

From the logistic regression model, we calculated hospital-level observed vs expected DNR ratios and visually assessed the variation in both unadjusted condition-specific hospital-level DNR prevalence and the observed vs expected value using density plots. This visualization allowed us to consider the extent of interhospital differences in DNR documentation.

### Association Between RSMR or RSRR and DNR Prevalence

Within each condition-specific cohort, we used the hospital-level RSMR or RSRR reported by the CMS as dependent variables in distinct inverse-variance–weighted linear regression models with the following independent variables: (1) quintiles of hospital-level DNR prevalence, with prevalence defined as the percentage of patients with POA DNR status; (2) mean hospital-level age modeled with a 5-knot restricted cubic spline function; and (3) mean hospital-level Elixhauser comorbidity index score modeled with a 5-knot restricted cubic spline function.

The inverse-variance weights were estimated from the CIs of the RSMR and RSRR in the Hospital Compare data, where SE = [(Upper CI – Lower CI) / (2 × 1.96)] and weight = 1 / SE.^2^

The adjusted least-squared mean RSMR and RSRR (and 95% CIs) for each quintile of hospital-level DNR prevalence were reported along with the overall Wald *P* value for DNR quintiles. We also compared the condition-specific mean RSMR and RSRR of the fifth (highest) quintiles with those of the first (lowest) quintiles using a Bonferroni-corrected *P* value. We conducted a sensitivity analysis using a more inclusive definition of DNR prevalence, in which patients were considered to have DNR status regardless of whether the DNR order was POA.

### Association Between DNR Prevalence and HRRP Penalty

To assess the association between hospital-level POA DNR status and HRRP penalty, we pooled patient-level data from the 4 readmission cohorts included in the HRRP penalty calculation and assessed hospital-level POA DNR status. Next, we used logistic regression, adjusting for age and Elixhauser comorbidity index score (using restricted cubic spline functions), to identify the association between POA DNR prevalence (independent variable) and avoidance of HRRP penalty (dependent variable).

### Statistical Analysis 

All analyses were performed with SAS, version 9.4 (SAS Institute Inc). Two-sided *P* < .05 indicated statistical significance. Data were analyzed from July 2019 to March 2020.

## Results

This study analyzed 4 884 237 inpatient encounters across the 5 condition-specific 30-day mortality cohorts (patient mean [SD] age, 78.8 [8.5] years; 2 608 182 women [53.4%] and 2 276 055 men [46.6%]) and 4 450 378 inpatient encounters across the 4 condition-specific 30-day readmission cohorts (patient mean [SD] age, 78.6 [8.5] years; 2 349 799 women [52.8%] and 2 100 579 men [47.2%]). Within the mortality cohorts, DNR status was POA in 790 988 encounters (16.2%) and present at any time in 946 391 encounters (19.4%). Within the readmission cohorts, DNR status was POA in 674 030 encounters (15.1%) and present at any time in 776 071 encounters (17.4%). Condition-specific patient-level characteristics are presented in Table 1.

**Table 1.** Patient-Level Characteristics of Condition-Specific Cohorts, 2015-2018

Characteristic	AMI	HF	Stroke	Pneumonia	COPD
**30-d Mortality cohorts (n = 4 884 237)**					
No. (%)	477 313 (9.8)	1 353 096 (27.7)	563 793 (11.5)	1 486 534 (30.4)	1 003 501 (20.5)
DNR status, No. (%)					
POA	55 470 (11.6)	220 177 (16.3)	90 027 (15.9)	300 661 (20.2)	124 653 (12.4)
Present at any time	70 835 (14.8)	261 657 (19.3)	111 611 (19.8)	353 865 (23.8)	148 423 (14.8)
Elixhauser comorbidity index score, median (IQR)	4 (–1 to 11)	7 (1 to 14)	3 (–1 to 10)	11 (5 to 19)	8 (0 to 14)
Age, median (IQR), y	77 (70 to 84)	80 (73 to 87)	79 (72 to 86)	80 (73 to 87)	75 (70 to 81)
Financial penalty possible?^a^	Yes	Yes	No	Yes	No
**30-d Readmission cohorts (n = 4 450 378)**					
No. (%)	549 259 (12.3)	1 431 177 (32.2)	NA	1 489 018 (33.5)	980 924 (22.0)
DNR status, No. (%)					
POA	53 741 (9.8)	221 539 (15.5)	NA	281 548 (18.9)	117 202 (11.9)
Present at any time	63 718 (11.6)	255 582 (17.9)	NA	321 607 (21.6)	135 164 (13.8)
Elixhauser comorbidity index score, median (IQR)	3 (–1 to 10)	6 (1 to 14)	NA	11 (4 to 19)	8 (0 to 14)
Age, median (IQR), y	76 (70 to 83)	80 (73 to 87)	NA	80 (72 to 87)	75 (70 to 81)
Financial penalty possible?^b^	Yes	Yes	NA	Yes	Yes

Abbreviations: AMI, acute myocardial infarction; COPD, chronic obstructive pulmonary disease; DNR, do not resuscitate; HF, heart failure; IQR, interquartile range; NA, not applicable; POA, present on admission.

^a^ Penalty through value-based purchasing.

^b^ Penalty through the Hospital Readmissions Reduction Program.

Hospital-level median (interquartile range [IQR]) prevalence of POA DNR status in the mortality cohorts varied: 11% (7%-16%) for AMI, 13% (7%-23%) for HF, 14% (9%-22%) for stroke, 17% (9%-26%) for pneumonia, and 10% (5%-18%) for COPD. For the readmission cohorts, the hospital-level median (IQR) POA DNR prevalence was 9% (6%-15%) for AMI, 12% (6%-22%) for HF, 16% (8%-24%) for pneumonia, and 9% (4%-17%) for COPD.

Across all condition-specific mortality cohorts, the Elixhauser comorbidity index score showed a statistically significant and generally nonlinear association with DNR status (eFigure 1 in the Supplement). Visual assessment indicated minimal potential systemic bias from lack of documentation of DNR status among patients at the extremes (sickest or healthiest).

Unadjusted hospital-level condition-specific DNR prevalence (Table 2) was highest in the pneumonia mortality cohort (median [IQR)], 17% [9%-26%]) and lowest in the COPD readmissions cohort (median [IQR], 9%; [4%-17%]). Condition-specific hospital-level characteristics are presented in Table 2. For example, in the AMI mortality cohort, the median (IQR) hospital-level Elixhauser comorbidity index score was 5.7 (4.8-6.7) and the median (IQR) age was 77 (76-79) years.

**Table 2.** Hospital-Level Characteristics of Condition-Specific Cohorts, 2015-2018

Characteristic	AMI	HF	Stroke	Pneumonia	COPD
**30-d Mortality cohorts **					
No.	2268	3488	2501	4009	3536
DNR status prevalence					
POA, median (IQR), %	11 (7-16)	13 (7-23)	14 (9-22)	17 (9-26)	10 (5-18)
Present at any time, median (IQR), %	14 (10-20)	16 (9-26)	18 (12-26)	21 (12-29)	13 (6-20)
Elixhauser comorbidity index, median (IQR)	5.7 (4.8-6.7)	7.5 (6.2-8.6)	5.1 (4.2-6.1)	11.7 (9.8-13.2)	8.0 (6.5-9.3)
Age, median (IQR), y	77 (76-79)	80 (79-82)	79 (78-80)	80 (79-81)	76 (75-77)
RSMR, median (IQR), %	12.8 (12.1-13.5)	11.6 (10.5-12.7)	13.7 (12.8-14.7)	15.6 (14.4-17.0)	8.4 (7.8-9.2)
POA DNR quintile cut points					
20th Percentile	6.0	5.5	7.7	6.5	3.8
40th Percentile	9.1	10.6	12.0	13.4	7.8
60th Percentile	12.6	16.5	17.1	20.0	12.6
80th Percentile	18.2	25.4	24.5	28.3	20.1
Observed vs expected POA DNR, median (IQR)	0.94 (0.61-1.38)	0.81 (0.41-1.37)	0.90 (0.56-1.38)	0.85 (0.43-1.29)	0.81 (0.37-1.42)
Financial penalty possible?^a^	Yes	Yes	No	Yes	No
**30-d Readmission cohorts **					
No.	2089	3564	NA	4010	3574
DNR status prevalence					
POA, median (IQR), %	9 (6-15)	12 (6-22)	NA	16 (8-24)	9 (4-17)
Present at any time, median (IQR), %	11 (7-17)	15 (8-24)	NA	19 (10-27)	11 (6-19)
Elixhauser comorbidity index score, median (IQR)	5.3 (4.3-6.4)	7.1 (5.8-8.1)	NA	11.2 (9.3-12.7)	7.8 (6.3-9.1)
Age, median (IQR), y	77 (76-79)	80 (79-82)	NA	80 (79-81)	76 (75-77)
RSRR, median (IQR), %	15.7 (15.1-16.3)	21.6 (20.7-22.6)	NA	16.5 (15.9-17.3)	19.4 (18.8-20.1)
POA DNR quintile cut points					
20th Percentile	4.8	4.7	NA	5.4	3.4
40th Percentile	7.7	9.7	NA	12.4	7.3
60th Percentile	11.1	15.5	NA	18.4	12.0
80th Percentile	17.0	24.3	NA	26.7	19.4
Observed vs expected POA DNR, median (IQR)	0.92 (0.57-1.43)	0.79 (0.38-1.36)	NA	0.83 (0.40-1.31)	0.80 (0.35-1.41)
Financial penalty possible?^b^	Yes	Yes	NA	Yes	Yes

Abbreviations: AMI, acute myocardial infarction; COPD, chronic obstructive pulmonary disease; DNR, do not resuscitate; HF, heart failure; IQR, interquartile range; NA, not applicable; POA, present on admission; RSMR, Risk-Standardized Mortality Rate; RSRR, Risk-Standardized Readmission Rate.

^a^ Penalty through value-based purchasing.

^b^ Penalty through the Hospital Readmissions Reduction Program.

Across all condition-specific 30-day mortality cohorts, variation was found in observed to expected DNR prevalence ratios (eFigure 2 in the Supplement), with a median (IQR) observed vs expected DNR prevalence ranging from 0.79 (0.38-1.36) in the HF readmission cohort to 0.94 (0.61-1.38) in the AMI mortality cohort (Table 2). The median (IQR) observed vs expected DNR prevalence ratios in the readmission cohorts were approximately normally distributed for AMI (0.94 [0.61-1.38]) and stroke (0.90 [0.56-1.38]) cohorts, whereas observed vs expected DNR prevalence ratios were skewed to the right for HF (0.81 [0.41-1.37]), COPD (0.81 [0.37-1.42]), and pneumonia (0.85 [0.43-1.29]).

**Figure 1.**
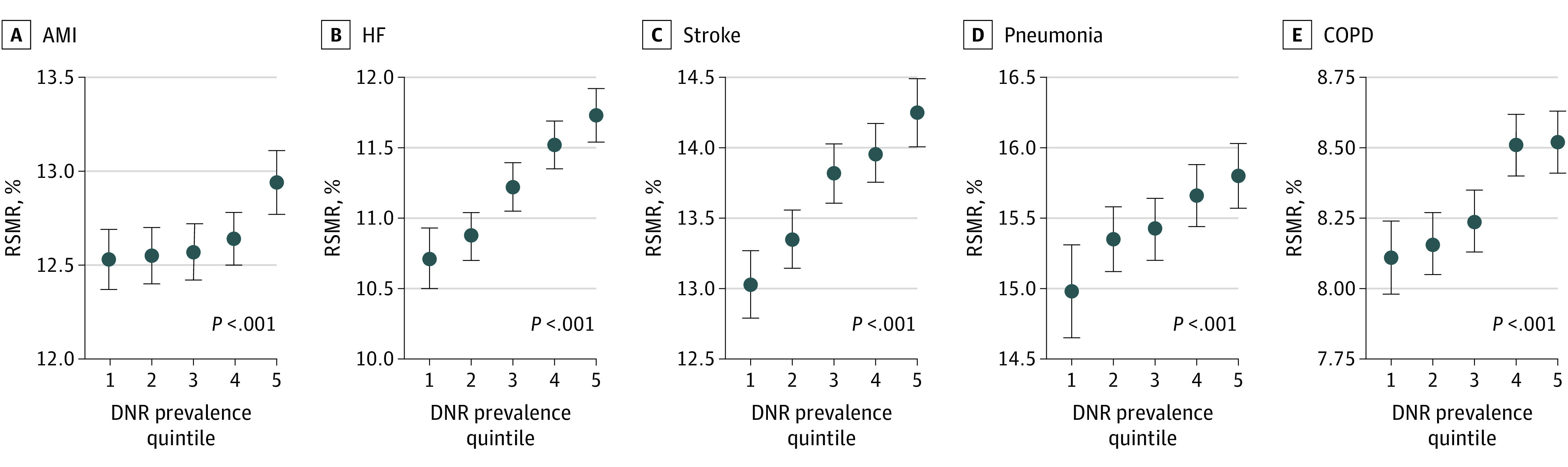
Elixhauser Comorbidity Index– and Age-Adjusted Association Between Hospital-Level Do Not Resuscitate (DNR) Prevalence Quintile and Risk-Standardized Mortality Rate (RSMR) 30-Day RSMRs were significantly higher for hospitals in the highest present-on-admission DNR prevalence quintiles vs the lowest quintiles (all *P* < .001). The points represent the Elixhauser Comorbidity Index- and age-adjusted 30 day least square mean for RSMR in each of the 5 DNR quartiles. The error bars represent the Wald 95% CIs. 1 Indicates the lowest quintile and 5 the highest quintile; AMI, acute myocardial infarction; COPD, chronic obstructive pulmonary disease; and HF, heart failure.

**Figure 2.**
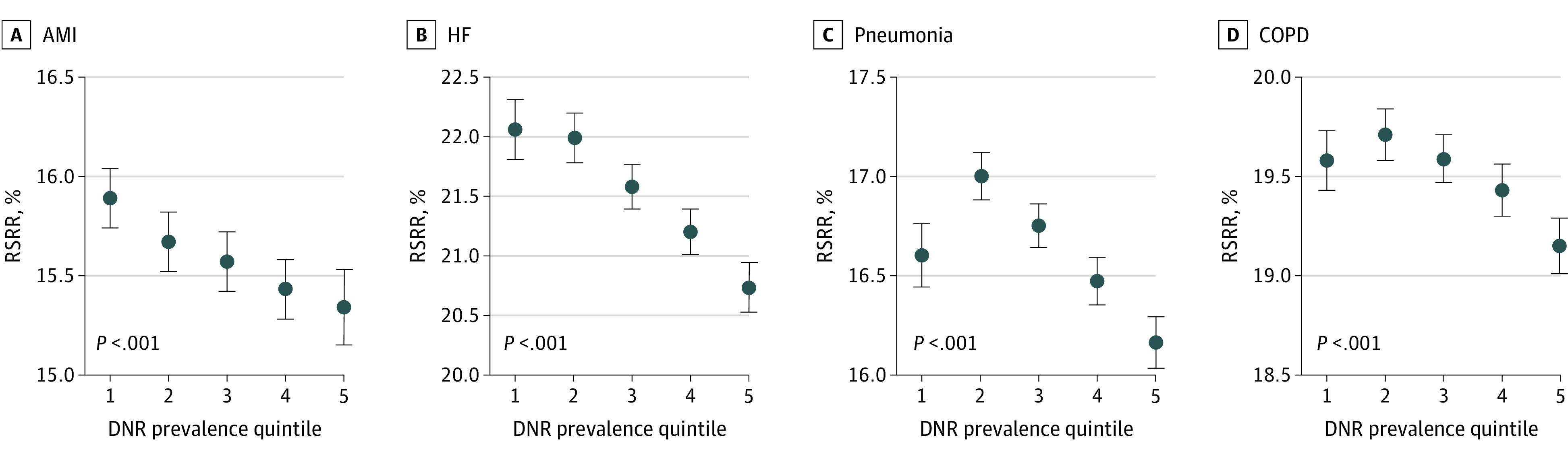
Elixhauser Comorbidity Index– and Age-Adjusted Association Between Hospital-Level Do Not Resuscitate (DNR) Prevalence Quintile and Risk-Standardized Readmission Rate (RSRR) The highest present-on-admission DNR prevalence quintiles had the lowest 30-day RSRR (all *P* < .001). The points represent the Elixhauser Comorbidity Index- and age-adjusted 30 day least square mean for RSRR in each of the 5 DNR quartiles. The error bars represent the Wald 95% CIs. 1 Indicates the lowest quintile and 5 the highest quintile; AMI indicates acute myocardial infarction; COPD, chronic obstructive pulmonary disease; and HF, heart failure.

In all condition-specific mortality cohorts, the RSMR was higher in the fifth DNR prevalence quintile vs the first quintile (AMI: 12.9 [95% CI, 12.8-13.1] vs 12.5 [95% CI, 12.4-12.7]; HF: 11.7 [95% CI, 11.5-11.9] vs 10.7 [95% CI, 10.5-10.9]; stroke: 14.0 [95% CI, 13.7-14.1] vs 13.0 [95% CI, 12.8-13.3]); pneumonia: 15.8 [95% CI, 15.6-16.0] vs 15.0 [95% CI, 14.7-15.3]; COPD: 8.5 [95% CI, 8.4-8.6] vs 8.1 [8.0-8.2]; all *P* < .001), and an association between RSMR and DNR prevalence quintile was found across all conditions (all Wald *P* < .001) (Figure 1). In all condition-specific readmission cohorts, the RSRR was lower in the fifth DNR prevalence quintile than in the first quintile (AMI: 15.3 [95% CI, 15.1-15.5] vs 15.9 [95% CI, 15.7-16.0]; HF: 20.7 [95% CI, 20.5-20.9] vs 22.1 [95% CI, 21.8-22.3]; pneumonia: 16.2 [95% CI, 16.0-16.3] vs 17.0 [95% CI, 16.9-17.1]; and COPD: 19.2 [95% CI, 19.0-19.3] vs 19.6 [95% CI, 19.4-19.7]; all *P* < .001), and an association between RSRR and DNR prevalence quintile was found across all conditions (all Wald *P* < .001) (Figure 2). The sensitivity analysis in which we removed the POA requirement for patients with DNR status showed similar results (eTable in the Supplement).

**Figure 3.**
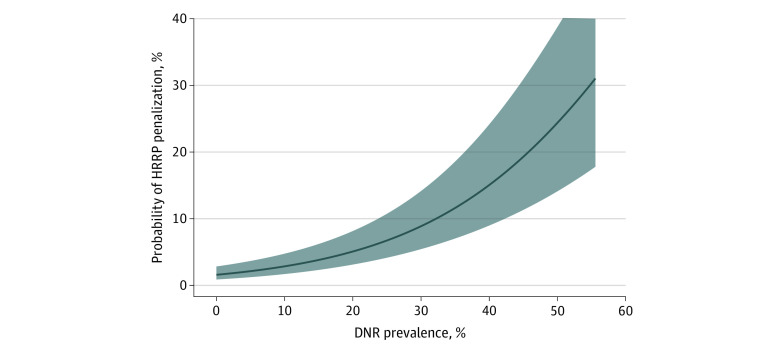
Elixhauser Comorbidity Index– and Age-Adjusted Association Between Hospital-Level Present-on-Admission Do Not Resuscitate (DNR) Prevalence and Probability of Hospital Readmissions Reduction Program (HRRP) Penalization

Among the 2948 hospitals included in the analysis of the association between hospital-level POA DNR prevalence and HRRP penalty, 200 (6.8%) hospitals avoided an HRRP penalty from fiscal years 2016 to 2018. After adjustment for age and Elixhauser comorbidity index score, POA DNR prevalence was associated with HRRP penalty avoidance (odds ratio per 1% absolute increase in POA DNR prevalence, 1.06; 95% CI, 1.04-1.08; *P* < .001) (Figure 3).

## Discussion

To our knowledge, this study is the first to use national Medicare claims data to assess the association between hospital-level prevalence of patients with POA DNR status and 30-day RSMRs and RSRRs across 5 conditions. A higher prevalence of patients with POA DNR status was associated with a higher RSMR. Conversely, a higher prevalence of patients with POA DNR status was associated with a lower RSRR. The 30-day RSMR and RSRR models do not adjust for POA DNR status, which may be a factor in unearned HRRP financial penalties and inaccurate CMS Overall Hospital Quality Star Ratings.

The association between hospital DNR case mixes and quality outcomes has been examined previously, notably by Walkey and colleagues.^22,23^ Specifically, Walkey and colleagues^22,23^ found that hospitals with higher rates of DNR prevalence were more likely to be outliers for pneumonia mortality, and these authors hypothesized that the lack of adjustment for DNR status was associated with biased penalization of hospitals that in reality were providing high-quality, patient-centered care.^22,23^ Results of the present analysis not only support this hypothesis and provide expanded evidence of this pattern across multiple conditions and quality outcomes linked with CMS reimbursements but also offer the first direct evidence of the implications of this association for HRRP penalties. In addition, these results are consistent with findings from 2 studies of approximately 300 hospitals in the California State Inpatient Database (CA-SID). The CA-SID study reported an increase in the C statistic from 0.75 to 0.78 when comparing a CMS-based risk-standardized AMI mortality model to an improved risk model that adjusted for DNR status^11^ and a decrease in readmission rates for patients with pneumonia who had a DNR status.^12^ In the AMI analysis, Bruckel and colleagues^11^ found that 6 of 25 high-mortality outlier hospitals were reclassified as nonoutliers under the improved DNR risk-standardized model. Likewise, both the hospital-level prevalence and variation of POA DNR status reported in the CA-SID study (unadjusted median [IQR] DNR prevalence: 8% [4%-14%]) were similar to those in the CMS claims data (Inpatient Standard Analytical File). This similarity in DNR rates between CMS claims data and the CA-SID study, which showed an 84% agreement between the claims-based DNR status variable and medical record abstraction,^24^ suggests that POA DNR status is captured in the CMS claims data at acceptable rates to permit its inclusion in risk-standardized models.

Patients with DNR status do not receive cardiopulmonary resuscitation during an acute event and are expected to have greater risks of 30-day mortality. Conversely, patients with DNR status are more likely to be discharged to hospice or end-of-life care, making 30-day readmission less likely.^12^ Under the current paradigm, hospitals that provide treatment to higher proportions of patients with complex conditions, terminal illness, and/or DNR status may be reported to have higher RSMRs and lower RSRRs than if an appropriate DNR adjustment was made.

The omission of DNR status as a risk-adjustment variable was attributed to concerns about its availability and documentation in claims data,^25^ including whether DNR status acquired during hospitalization represented a preventable complication or natural history of disease. However, the same rationale exists for many of the clinical risk factors in the CMS models, and the variation may reflect true prevalence or accuracy of documentation. One example from the CMS models is protein-calorie malnutrition, a comorbidity for which reported hospital prevalence may reflect documentation rather than case mix.^26^ Furthermore, no evidence is available to suggest that hospitals could take advantage of the system by overdocumenting DNR status. Abusing the system for DNR status is less possible than for other comorbidities in the CMS models because, unlike clinical risk factors, the documentation of DNR status requires the additional burden of obtaining a patient’s legal consent and signature and/or even the presence of a witness in some jurisdictions. Conversely, no signatures or witnesses are needed for a physician to document risk factors, such as protein-calorie malnutrition.

This analysis suggested that there are implications for HRRP penalization if POA DNR status was not controlled for during risk standardization. Specifically, for 2 hospitals of equivalent age and comorbidity case mixes among patients in the readmission cohorts, a hospital with a 10% greater absolute prevalence of POA DNR status (eg: 25% vs 15%) has nearly 2-fold better odds of avoiding HRRP penalization, implying that hospitals with low DNR prevalence could receive either a lower penalty or no penalty if POA DNR status were accounted for in risk standardization. Conversely, hospitals with high DNR prevalence could receive a larger HRRP penalty if POA DNR status were included in risk standardization.

We did not assess the association of lower readmissions and higher mortality with value-based purchasing payments in hospitals with high DNR prevalence. Thus, although hospitals with high DNR prevalence may have an advantage under HRRP owing to the lack of risk adjustment for DNR status, it is possible that the reverse is true under value-based purchasing when the opposing effects of 30-day mortality are considered.

### Strengths and Limitations

This study has some strengths. It used a large national sample of CMS claims data from more than 4000 US hospitals. It also closely adhered to the CMS condition-specific cohort inclusion and exclusion criteria.

This study has some limitations. First, it had an ecological design, which makes it possible that patients with POA DNR status were not direct factors in the increased mortality rates at hospitals with high DNR prevalence. However, given that patients with a DNR status are known to have greater mortality risks, there is strong reason to suspect that these patients may be direct contributors to higher mortality rates. Second, some of the variation in DNR status may be associated with interinstitutional coding differences, physician preferences and comfort with discussing DNR status, and patient preferences and values. Given these differences, misclassification of DNR status may have occurred that could have biased the results to the null, particularly among several hospitals with 0% reported DNR prevalence. Third, although the hospital-level DNR prevalence rates in this study were similar to those in the CA-SID study,^11^ which was validated with medical record reviews, we did not validate DNR documentation in this analysis. Furthermore, the inclusion of hospice patients may have introduced bias because these patients were excluded from the CMS metrics. Nevertheless, less than 2% of all encounters were hospice exclusions under the CMS method, so it is unlikely that this small percentage of encounters changed the results.^14^ We did not assess the association between in-hospital DNR status acquisition and RSMR or RSRR, which could be an important area for future research given the increasing consideration of patient-centered palliative care in the hospital setting. Fourth, socioeconomic and demographic factors were likely associated with patients’ DNR status. We did not adjust for these factors in these analyses, but they may help explain the associations between hospital DNR case mixes and RSMR or RSRR.

## Conclusions

Under the current CMS risk-standardization models for 30-day mortality and readmission, mortality rates appeared to be worse among hospitals with greater proportions of patients with POA DNR status, whereas readmission rates appeared to be better among hospitals with greater proportions of patients with POA DNR status. Findings from this study suggest that controlling for DNR status would improve estimates for 30-day RSMRs and RSRRs and would have direct implications for HRRP financial penalty and hospital performance rankings.

## Notes

Supplement. **eTable.** Sensitivity Analysis: Results From Expanding DNR Definition to Include DNR At Any Time During Inpatient Stay Versus the Main Analysis (POA DNRs Only) **eFigure 1.** Patient-Level 30-Day Mortality Condition-Specific Age-Adjusted Associations Between Elixhauser Index and Probability of Having a Present-on-Admission DNR **eFigure 2.** Hospital-Level 30-Day Mortality Condition-Specific Distributions of Observed Present-on-Admission DNR and Observed/Expected Present-on-Admission DNR
