# Supplementary material for: Association of Do-Not-Resuscitate Patient Case Mix With Publicly Reported Risk-Standardized Hospital Mortality and Readmission Rates
Source: JAMA Netw Open. 2020 Jul 14;3(7):e2010383. doi: 10.1001/jamanetworkopen.2020.10383 (PMC7361656; doi:10.1001/jamanetworkopen.2020.10383)
Supplement: Supplement. — eTable. Sensitivity Analysis: Results From Expanding DNR Definition to Include DNR At Any Time During Inpatient Stay Versus the Main Analysis (POA DNRs Only) eFigure 1. Patient-Level 30-Day Mortality Condition-Specific Age-Adjusted Associations Between Elixhauser Index and Probability of Having a Present-on-Admission DNR eFigure 2. Hospital-Level 30-Day Mortality Condition-Specific Distributions of Observed Present-on-Admission DNR and Observed/Expected Present-on-Admission DNR [file jamanetwopen-3-e2010383-s001.pdf]

Supplementary Online Content

Pollock BD, Herrin J, Neville MR, et al. Association of do-not-resuscitate patient case mix with publicly reported risk-standardized hospital mortality and readmission rates. *JAMA Netw Open.* 2020;3(7):e2010383. doi:10.1001/jamanetworkopen.2020.10383

eTable. Sensitivity Analysis: Results from Expanding DNR Definition to Include DNR At Any Time During Inpatient Stay Versus the Main Analysis (POA DNRs Only)

eFigure 1. Patient-Level 30-Day Mortality Condition-Specific Age-Adjusted Associations Between Elixhauser Index and Probability of Having a Present-on-Admission DNR

eFigure 2. Hospital-Level 30-Day Mortality Condition-Specific Distributions of Observed Present-on-Admission DNR and Observed/Expected Present-on-Admission DNR

This supplementary material has been provided by the authors to give readers additional information about their work.

©2020 Pollock BD et al. *JAMA Network Open*

eTable. Sensitivity Analysis: Results from Expanding DNR Definition to Include DNR At Any Time During Inpatient Stay Versus the Main Analysis (POA DNRs Only)

**30-day mortality cohorts**	**AMI**		**HF**		**Stroke**		**Pneumonia**		**COPD**	
Analysis	*Main*	*Sensitivity*	*Main*	*Sensitivity*	*Main*	*Sensitivity*	*Main*	*Sensitivity*	*Main*	*Sensitivity*
DNR prevalence	*12%*	*15%*	*16%*	*19%*	*16%*	*20%*	*20%*	*24%*	*12%*	*15%*
1st quintile, mean 30-day RSMR	12.53	12.62	10.71	11.32	13.03	13.23	14.98	15.56	8.11	8.29
2nd quintile, mean 30-day RSMR	12.55	12.69	10.87	11.33	13.35	13.49	15.35	15.58	8.16	8.37
3rd quintile, mean 30-day RSMR	12.57	12.82	11.22	11.57	13.82	13.91	15.42	15.76	8.24	8.49
4th quintile, mean 30-day RSMR	12.64	12.88	11.52	11.85	13.96	14.15	15.66	16.02	8.51	8.76
5th quintile, mean 30-day RSMR	12.94	13.17	11.73	12.10	14.25	14.43	15.80	16.00	8.52	8.78

**30-day readmission cohorts**	**AMI**		**HF**		**Stroke**		**Pneumonia**		**COPD**	
Analysis	*Main*	*Sensitivity*	*Main*	*Sensitivity*	-	-	*Main*	*Sensitivity*	*Main*	*Sensitivity*
DNR prevalence	*10%*	*12%*	*15%*	*18%*	-	-	*19%*	*22%*	*12%*	*14%*
1st quintile, mean 30-day RSRR	15.89	15.97	22.06	22.03	-	-	16.60	16.69	19.58	19.60
2nd quintile, mean 30-day RSRR	15.67	15.81	21.99	22.09	-	-	17.00	16.97	19.71	19.67
3rd quintile, mean 30-day RSRR	15.57	15.69	21.58	21.67	-	-	16.75	16.74	19.59	19.58
4th quintile, mean 30-day RSRR	15.43	15.57	21.20	21.37	-	-	16.47	16.50	19.43	19.47
5th quintile, mean 30-day RSRR	15.34	15.48	20.73	20.99	-	-	16.16	16.24	19.15	19.19

©2020 Pollock BD et al. *JAMA Network Open*

eFigure 1. Patient-Level 30-Day Mortality Condition-Specific Age-Adjusted Associations Between Elixhauser Index and Probability of Having a Present-on-Admission DNR

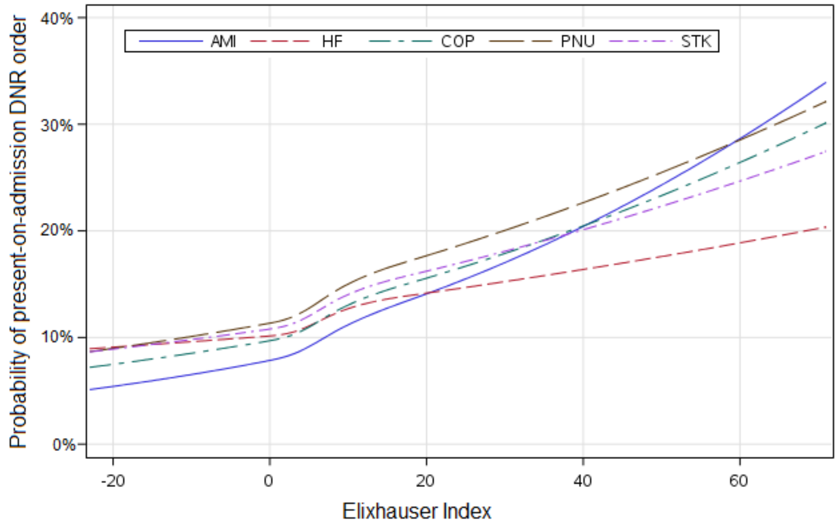

AMI=Acute Myocardial Infarction; HF=Heart Failure; COP=Chronic Obstructive Pulmonary Disease; PNU=Pneumonia; STK=Stroke; DNR=Do-Not-Resuscitate.

Elixhauser Index is a single integer value ranging from -32 (lowest possible comorbidity burden) to 99 (highest possible comorbidity burden); Modeled using a restricted cubic spline function from a pooled cohort of 4,884,237 condition-specific inpatient encounters among US Medicare beneficiaries from July 2015-June 2018.

©2020 Pollock BD et al. *JAMA Network Open*

eFigure 2. Hospital-Level 30-Day Mortality Condition-Specific Distributions of Observed Present-on-Admission DNR and Observed/Expected Present-on-Admission DNR

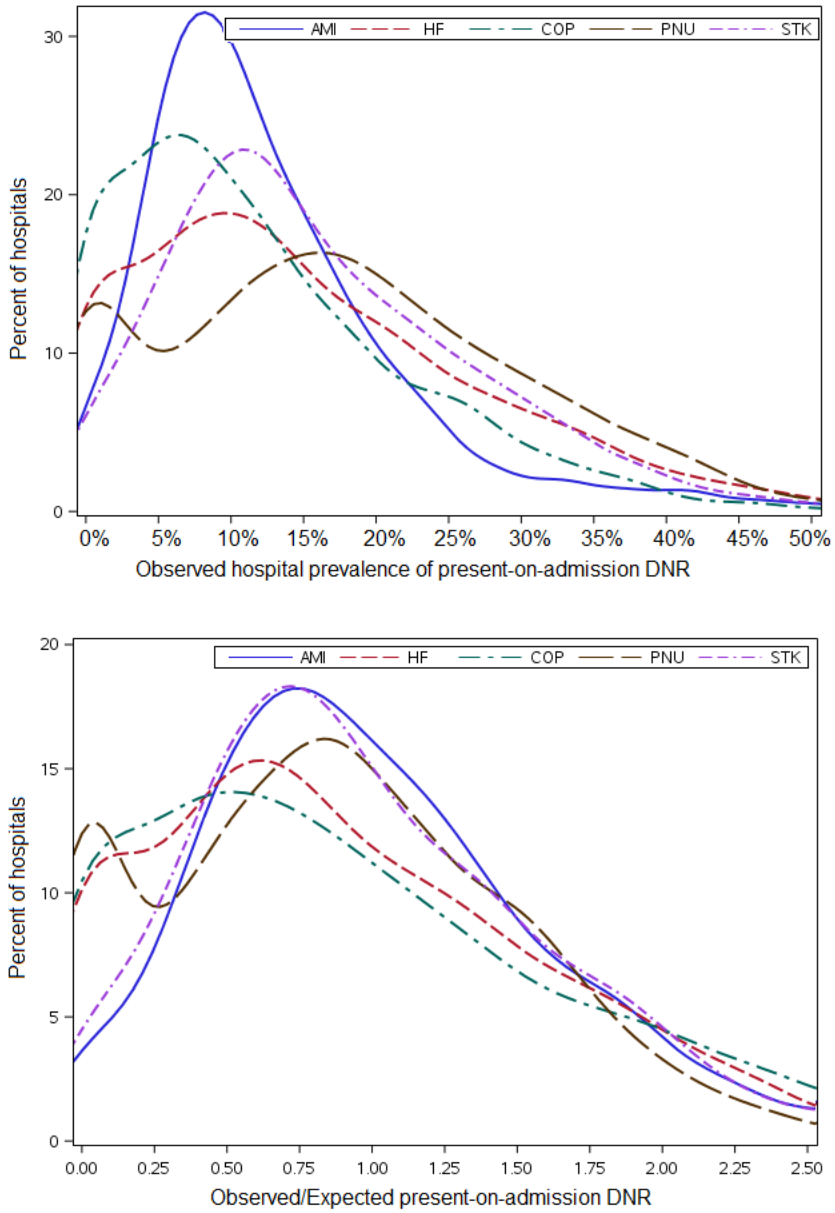

AMI=Acute Myocardial Infarction; HF=Heart Failure; COP=Chronic Obstructive Pulmonary Disease; PNU=Pneumonia; STK=Stroke; DNR=Do-Not-Resuscitate.

Density plots represent all US hospitals with a publicly reported 30-day risk-standardized mortality rate (on CMS Hospital Compare website, accessed August 2019) for AMI (n=2,268 hospitals); HF (n=3,488); stroke (n=2,501); pneumonia (n=4,009); COPD (n=3,536). The y-axis indicates percent of all hospitals.

©2020 Pollock BD et al. *JAMA Network Open*
